# A Comprehensive Overview of Pediatric Neoplasms at the Craniocervical Junction: Meningiomas, Schwannomas, and Chordomas

**DOI:** 10.7759/cureus.31083

**Published:** 2022-11-04

**Authors:** Brian Fiani, Ryan Jarrah, Jennifer Shields, Sulaman Durrani, Nicholas Panico, William Mualem, Karim Rizwan Nathani, Kory Pasko

**Affiliations:** 1 Neurosurgery, Weill Cornell Medical Center/NewYork-Presbyterian Hospital, New York, USA; 2 Neurosurgery, Mayo Clinic, Rochester, USA; 3 College of Human Medicine, Michigan State University, East Lansing, USA; 4 College of Osteopathic Medicine, Lake Erie College of Osteopathic Medicine, Erie, USA; 5 School of Medicine, Georgetown University, Washington, DC, USA

**Keywords:** chordoma, schwannoma, meningioma, pediatric oncology, craniocervical junction

## Abstract

Tumors of the craniocervical junction (CCJ) are complicated pathologies with high patient mortality or low quality of life. In the pediatric population, these tumors are less prevalent, with various symptomatic presentations that include motor and neurological manifestations. Three of the most common neoplasms at the CCJ in children are meningiomas, schwannomas, and chordomas. In this review, we will characterize the tissue biomarkers, clinical presentation, treatment methods, and surgical outcomes for these pediatric tumors at the CCJ. A comprehensive literature review was used using the PubMed Database. Keywords used were “craniocervical junction”, “pediatric”, “meningiomas”, schwannomas”, and “meningiomas”. Articles that were not related to the CCJ, included only adult cases, and non-English studies were filtered. Our search yielded a total of 11 studies, with a total of 239 pediatric patients with tumors at the CCJ. These studies were broken down as five for meningiomas, one for schwannomas, and eight for chordomas. In conclusion, resection of pediatric neoplasms at the CCJ is challenging due to anatomical limitations and the size of the patient. Within the CCJ, chordomas were the most prevalent tumor type, with schwannomas being the least prevalent. Literature findings indicate that genetic mutations of the NF2 gene associated with neurofibromatosis type II, as well as incomplete tumor resection, are predictors of poor outcomes. Further developments of monoclonal antibody chemotherapy and endoscopic approaches could expand treatment options for aggressive pediatric neoplasms at the skull base.

## Introduction and background

The craniocervical junction (CCJ) is a complex anatomical region composed of bony, neural, vascular, lymphatic, and supportive structures that change over the course of pediatric development. Due to the anatomic complexity and low incidence, treatment of tumors localized to the skull base within the CCJ in pediatric populations pose unique treatment challenges to neurosurgeons and the rest of the care team. Advances in the understanding of pediatric neoplasm growth, imaging detection modalities, and treatment options in this region have led to reductions in postoperative morbidity and mortality. However, treatment of these tumors is still associated with a significant risk of recurrence, postoperative complications, and mortality [1]. The use of multidisciplinary team-based care and referral to specialized treatment centers when necessary is encouraged in order to further optimize treatment outcomes. Nonsurgical treatment strategies consist of stereotactic radiosurgery, radiation, and primary or adjuvant chemotherapeutic agents [2]. When tumors are refractory to nonoperative treatment, microsurgical or wide-based resection is often indicated.

The bony structures of the CCJ include the occipital skull base, atlas, and axis. The articulation between the CCJ and the subaxial cervical spine gives rise to complex movement of the head while simultaneously housing and protecting vital neurovascular structures. Postoperative instability of the CCJ increases the risk of catastrophic consequences, such as nerve or arterial compression and noncommunicating hydrocephalus [3]. Therefore, appropriate execution of indicated skull base surgical approaches is required when resecting tumors of this region in order to maintain postoperative stability of the CCJ and preserve the function of these vital neurovascular structures.

Neoplasms that arise within the CCJ originate from osseous structures, soft tissue, and neural tissue [4]. While the overall incidence of pediatric CCJ neoplasms localizing to the skull base is low, some of the most frequently occurring tumors include meningiomas, schwannomas, and chordomas. Meningiomas comprise approximately 5% of pediatric intracranial tumors with 35.8% of these meningiomas localizing to the skull base [5,6]. Schwannomas arise from various cranial and spinal nerves within the skull comprising approximately 5-10% of all pediatric intracranial tumors, a small subset of which localize to the skull base [7]. Approximately 54% of chordomas, tumors that arise from tissue with embryological notochord origin, are located within the cranium in pediatric populations, a small subset of which reside within the CCJ [4,8,9]. In this article, we will review the biomarkers of these tumors, treatment strategies, and surgical approaches, as well as surgical outcomes and complications of meningiomas, schwannomas, and chordomas in the pediatric skull base.

## Review

Pathophysiology and biomarker analysis

**Figure 1:**
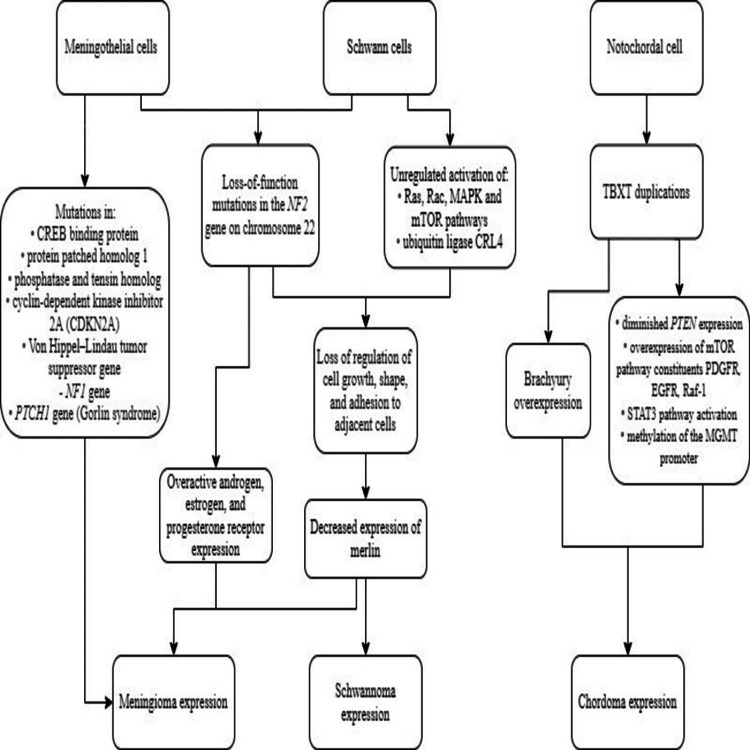
Genetic pathophysiology of pediatric meningiomas, schwannomas, and chordomas. Image credit: Authors Ryan Jarrah and Brian Fiani

Meningiomas normally originate from meningothelial cells of the arachnoid mater with corresponding dural attachment and are present anywhere along the exterior brain and ventricular system. Most meningioma mutations are sporadic with associated chromosomal deletions with some associated with genetic predispositions [10]. Neurofibromatosis type II (NF2) gene mutations are found in 50-60% of all sporadic meningiomas [10] (Figure 1). Sporadic gene mutations in AKT1, SMO, and KLF4 have been found with higher-grade meningiomas correlated with homozygous cyclin-dependent kinase inhibitor 2A (CDKN2A) gene deletions and telomerase reverse transcriptase (TERT)-promoter mutations [10]. Moreover, meningiomas can further present histopathological and immunological obscurities that can create a diagnostic challenge. Syncytial cells, nuclear clearing, and psammoma bodies are common histological features in meningiomas, yet present differently in subtypes [11]. Although immunochemistry markers such as cytokeratin 18 (CK18), somatostatin receptor 2A (SSTR2A), and epithelial membrane antigen (EMA) can confirm a meningioma, the variable expressivity among subtypes makes immunotherapies challenging for treating meningiomas [11].

Schwannomas arise from Schwann cells in peripheral nerves and have the highest genetic association with NF2, with vestibular schwannomas being a hallmark of the disease. In NF2 and many sporadic schwannomas, loss-of-function mutations in the NF2 gene on chromosome 22 lead to decreased expression of the protein merlin. In highly proliferative cellular environments, Schwann cell-mediated dephosphorylation of merlin induces cessation of tumor proliferation [12]. A lack of merlin expression, as well as its dephosphorylation, allow schwannoma development to proceed uninhibitedly. When assessing immunohistochemistry, complete loss of SOX10, neurofibromin, or p16 expression have been to have high sensitivities to differentiating for schwannoma growth. However, much is still unknown at the cellular and subcellar level for schwannomas. The degree of macrophage infiltration, activation of various pro-inflammatory pathways, and degree of cytokine expression have made the microenvironment of schwannoma proliferation quite complex to predict [13]. This microenvironment, along with the variable growth patterns of schwannomas, has complicated the testing of candidate immunomodulation agents for schwannomas [14].

Chordomas are benign primary tumors of soft tissue and bone that arise from embryological remnants of the primitive notochord [3]. The most common mutation in chordomas is of the TBXT gene. The TBXT gene encodes for a protein called brachyury, a transcription factor involved in notochordal growth. An excess of brachyury has been found to contribute to chordoma development; however, the exact mechanism remains unclear. Not all chordomas have TBXT gene alterations. Some have been associated with diminished PTEN expression; overexpression of mTOR pathway constituents PDGFR, EGFR, and Raf-1; STAT3 pathway activation; and methylation of the MGMT promoter in recurring chordomas of the clivus [15]. Histologically, the presentation of a chordoma is similar to that of a chondrosarcoma, presenting differentiating diagnostic challenges [16]. There is also a lack of epithelial biomarkers for chordomas, yet immunological markers such as higher CD8 expression, prevalence of PD-L1 immune cells, and higher vascular density, and the transcription factor brachyury were associated to have prognostic value [16]. However, the lack of understanding on the chordoma microenvironment, along with the slow-growing behavior of the tumor, has hindered advancement of robust immunotherapy treatment for chordomas [17].

Pediatric meningiomas

Clinical Presentation

**Figure 2:**
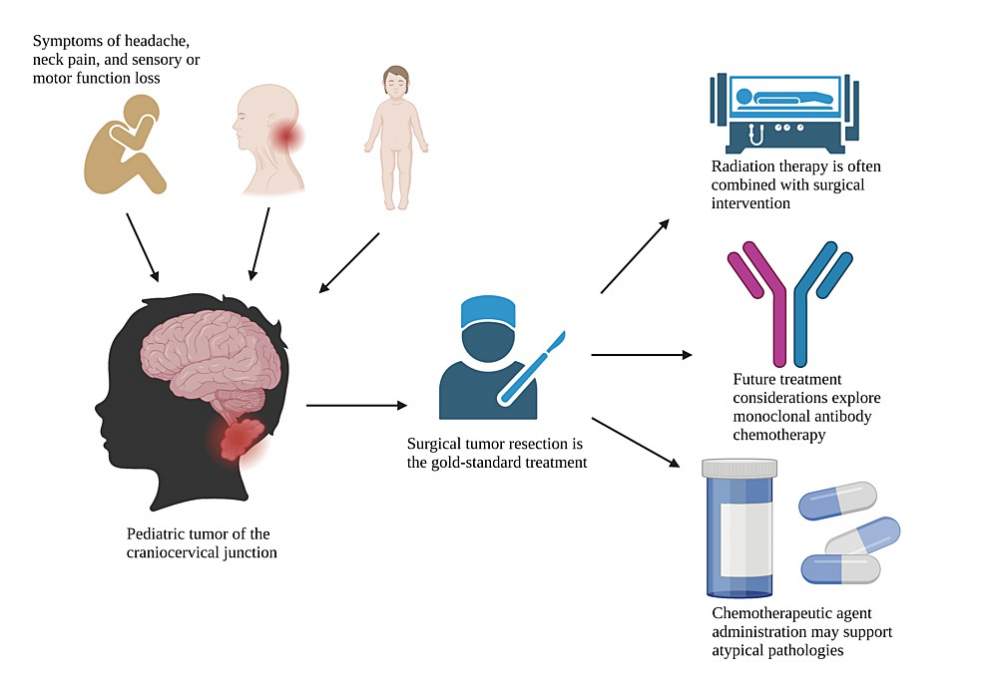
Clinical work-up and potential treatment pathways for pediatric tumors at the craniocervical junction. Image credit: Authors Ryan Jarrah and Brian Fiani

Meningiomas account for 37.6% of benign central nervous system tumors and 50% of benign brain tumors [5]. Pediatric meningiomas are even rarer, accounting for <5% of pediatric intracranial tumors and <2% of all meningiomas [5]. Due to its scarcity, literature offers limited knowledge for its development, course, and ideal management [18]. Meningiomas in children have been reported to have a relatively higher ratio of WHO grade II and III tumors, local invasion, and recurrence rates [19]. It is commonly associated with neurofibromatosis and history of ionizing radiation exposure [19]. The primary symptoms include increased intracranial pressure, as well as visual and motor disturbances. Contrary to its adult counterpart, pediatric meningiomas do not observe higher female prevalence but, rather, express a male predominance [18,19]. Although rare, pediatric meningiomas at the CCJ pose a serious challenge for surgeons due to the pathology and complexity of the local anatomy [5]. Similar to adults, total surgical resection is the recommended treatment for meningiomas in children [5,6,18,19] (Figure 2).

Treatment

Meningioma surgical treatment should be carefully planned with preoperative pediatric anatomic considerations to avoid serious surgical complications. For example, due to forward displacement of pterion in children, a standard burr hole can penetrate the periorbita rather than the anterior fossa [5]. When appropriate, minimally invasive approaches such as transsphenoidal and endoscopic procedures can allow the avoidance of facial growth plates and developing dentition [5,20,21]. For posterior cranial base surgery, there are multiple considerations in the pediatric population, including immature mastoid cell aeration. Due to immature aeration in pediatric patients, drilling of this region can be impeded by the dense bone, as well as make identification of labyrinths difficult. However, this can be circumvented. The literature reports several tumor resection approaches, including posterior petrosal, translabyrinthine, retrosigmoid, transcochlear, retrolabyrinthine, transjugular, and transcondylar [5,22,23]. The posterior petrosal technique was found to preserve auditory and facial nerve function in pediatric meningiomas [24]. The transcochlear passage is associated with a higher risk of facial palsy due to intraoperative complications compared with translabyrinthine or retrosigmoid approaches [5]. Although pediatric CCJ meningiomas represent a small subset of pediatric intracranial oncology, they require thorough preoperative planning for optimal resection of the pathology. A multidisciplinary approach can be observed to accommodate considerations for the immature skull and optimal skull base surgical technique (Table 1).

**Table 1:** Overview of studies investigating craniocervical junction (CCJ) tumors in pediatric patients.

Author:	Study Type:	Sample Size:	Tumor Type:	Tumor Location	Major Findings:
Menezes et al., 2008 [4]	Case Series	38	Multiple Tumors	CCJ	Chordomas were the most prevalent tumor type at the CCJ (n=8). Headache and neck pain were the most common symptoms. There were two cases of pediatric schwannomas at the upper cervical region
Athanasiou et al., 2015 [24]	Case Report	1	Meningioma	CCJ	Patient presented with gait instability and tetraparesis. CCJ tumors may have an intradural and extradural presentation.
Pinto et al., 2012 [25]	Retrospective chart review	24	Meningioma	CCJ	Seven patients had NF2, with three out of these seven in the CCJ.
Miranda et al., 2009 [26]	Retrospective chart review	1	Meningioma	CCJ	Radiographic evidence of craniospinal tumoral dissemination may not be prevalent.
Bai et al., 2020 [27]	Retrospective chart review	62	Chordomas	CCJ	The CCJ was the most common location of tumor occurrence, with the highest proportion of large tumors, yet lowest gross resection rate.
Sebro et al., 2016 [28]	Retrospective chart review	31	Chordomas	CCJ	72% of the 56 pediatric cases were found at the CCJ. Pediatric patients with CCJ chordomas had lower mortality than the adult cohort.
Borba et al., 1996 [29]	Literature review	79	Chordomas	CCJ	Many cases of chordomas at the CCJ have an atypical histological pattern.
Igaki et al., 2004 [30]	Case report	1	Chordomas	CCJ	Treatment with high-proton therapy was successful for 9 years, prorated follow-up in excess of 10 years is warranted.
D’Ortenzio et al., 2021 [31]	Case report	1	Chordomas	CCJ	CT-guided transoral biopsy may be a valuable less invasive method for craniocervical masses.
Eco et al., 2019 [32]	Case report	1	Chordomas	CCJ	The use of an expandable cage for reconstruction of the anterior CCJ may be a valuable method for reducing extensive dissection for tumor resection.
Ahmed et al., 2008 [33]	Retrospective chart review	750	NA	CCJ	A chordoma is a predictor for instability after CCJ fusion.

Literature Outcomes

Menezes et al. conducted an analysis of pediatric CCJ tumors. Out of 38 children, 5 had meningiomas, making them the second most prevalent tumor type [4]. In a further study by Pinto et al., 24 pediatric patients with meningiomas had radiological data taken to analyze histopathological or radiographic indicators. The median age was 14.3, with an equal number of males to females (n=12) [25]. Eight patients had multiple meningiomas. Seven patients had a known diagnosis of NF2, with three of these seven being located at the craniocervical junction. It was noted that a diagnosis of NF2 led to more tumors being located in unusual locations such as the CCJ. [25]. 

Pediatric schwannomas

Clinical Presentation

Schwannomas are the most common benign nerve sheath tumor with an incidence of about one for a population of 100,000 and represent about 5-10% of all intracranial tumors [7,34]. Schwannomas located at the CCJ are rare, with the clinical presentation variable and sometimes in association with NF2 [8]. Symptoms may only arise once the tumor has grown significantly in size due to the large subarachnoid spaces of the cervicomedullary junction [4]. Schwannomas of the skull base can arise from any cranial nerve or can emanate from spinal nerves; examples include nerves of the jugular foramen, hypoglossal nerve, and C1 and C2 spinal nerves. In the craniocervical junction, schwannomas are less frequently encountered than meningiomas, chordomas, and paragangliomas [35]. Schwannomas in children may appear as part of the neurocutaneous disorder neurofibromatosis type I and tend to become symptomatic with multiple or bilateral tumors. In a database analysis of the University of Iowa Hospitals and Clinics Neurosurgery Craniocervical Junction Registry, of 808 patients evaluated between 1977 and 2003, it was found that the average age at which schwannomas of the CCJ present is 38 years old and predominantly occurring in males [36]. Most patients (63%) experience pain as the major symptom, with paresthesias appearing in a quarter of cases and lower cranial nerve deficits developing in about 38% of patients or myelopathy in 50% [36]. Most complications associated with these tumors include those related to lower cranial nerve neuropathies, commonly resulting in hoarseness, swallowing difficulties, or dyspnea.

Treatment

During planning of the surgical approach to the region of the CCJ, it is imperative to consider the medulla and spinal cord, the lower cranial and upper spinal nerves, the vertebral arteries with their branches, the veins along with the dural sinuses, and the ligaments and muscles uniting the atlas, axis, and occipital bone [37,38]. Surgical removal is the treatment of choice and curative when complete excision is obtained [37,38]. The far-lateral approach, including its variants the transcondylar, supracondylar, and paracondylar, is most commonly used when removing schwannomas of the CCJ. When a tumor was ventral to the dentate ligament, the ligament or the spinal accessory nerve was sectioned to obtain adequate exposure. When a dumbbell schwannoma appears, it can cause craniovertebral instability, which must be kept in mind during reconstruction [39,40].

Literature Outcomes

Outcomes describing pediatric CCJ schwannomas are very limited. In the study on CCJ pediatric tumors by Menezes et al., two of the tumors were schwannomas, one localized to the foramen magnum and the other to the C2 spinal nerve [4]. The patient presented with severe left arm and neck pain that was aggravated by movement, along with spastic quadriparas. There was no indication of neurofibromatosis, but imaging concluded findings of an intradural and extradural schwannoma with distortion of the vertebral artery. The patient was treated with a posterolateral surgical approach, with an uneventful intraoperative and postoperative course.

Pediatric chordomas

Clinical Presentation

Chordomas are slow-growing infiltrative tumors that arise from remnants of notochordal origin [41]. These are most commonly in the clivus, followed by the sacrum, and are usually rare with only 0.5 incidence per million patients, accounting for 1-8% of malignant bone tumors and 20% of spinal tumors [42,43]. With respect to the pediatric population, the mean age of diagnosis is 10 years with less than 5% of diagnoses presenting in the first two decades of life [42,43]. The earliest reported diagnosis is a clival chordoma in a neonate [42, 43]. Up to 54% of pediatric chordomas are intracranial and often located at the spheno-occipital synchondrosis, characteristically causing local clival destruction and neural compression [8,9]. Those at the CCJ predominantly present with pain and rigidity, as well as spinal cord compression if there is posterior enlargement as opposed to dysphagia and respiratory compromise with anterior enlargement [44].

Chordomas have the highest rate of presentation in adults between the ages of 40 and 70, with males being affected twice as much as females, with Caucasians being the most impacted [45]. About 5% of chordomas occur in children, and the demographics differ from their adult counterparts [4,9]. Pediatric chordomas are more prevalent in the female population, with the African American and Hispanic populations more commonly affected [4, 9]. Similar to adults, pediatric intracranial chordomas occur mostly at the clivus but have been found to be predominantly extraosseous, whereas intraosseous chordomas account for the majority of adult presentations [28].

Treatment

There are multiple surgical approaches to chordomas found in the CCJ. These include but are not limited to midline transoral, transmaxillary, transmandibular, high anterolateral retropharyngeal, upper cervical lateral, lateral petrous, and subtemporal approaches. More recently, less invasive approaches, such as conventional and expanded endoscopic endonasal approaches have been explored [46,47]. Skull base chordomas can often invade the CCJ, affecting the hypoglossal nerve between the occipital condyle and jugular tubercle. Such invasion makes gross total resection more difficult with increased risks for spinal instability, specifically craniocervical with special attention to the anterior arch of C1, and neurovascular injury [48,49]. Chordomas are generally not radiosensitive tumors, with previous literature noting that the minimum effective dose was more than 65-70 Gy, a level of radiation with high risk to surrounding structures [50]. While proton beam radiation therapy has previously shown a 46% control rate at five years, most studies have shown a limited use of radiation therapy in chordomas [51]. Recent advances in modern radiation therapy have revealed, although controversial, that there may be benefit with perioperative radiation therapy utilized as an adjunct [52]. Moreover, promising clinical trials have shown monoclonal antibody chemotherapy to have some efficacy. However, further research is needed to understand their role in clinical practice and guidelines. The current gold standard is surgery with the primary goal of gross total resection followed by separation surgery away from any essential neurovascular structures for any residual tumor to maximize adjunctive radiotherapy [41]. Chordomas with an anterior focus, however, have shown better outcomes in recent literature with endoscopic endonasal approaches, providing a minimally invasive approach to these select group of patients [46,47]. Nonetheless, surgical approach for CCJ chordomas must be individualized and tailored for each patient while keeping in mind to maximize resection at first presentation and minimizing morbidity.

Literature Outcomes

In the study by Menezes et al., chordomas were the most prevalent neoplasm at the CCJ with eight cases. Isolated nerve palsy was the most common finding for these chordoma patients. Literature findings demonstrate that chordomas near CCJ anatomical structures such as the clivus or foramen magnum have a varying prevalence. For example, Borba et al. conducted a literature review and found that out of 79 pediatric chordomas, 21 had chordomas affecting the clivus or spheno-occipital region, with 65% having an atypical histological pattern. All these cases had fatal outcomes by the time of their last follow-up [29]. A study by Bai et al. found 62 pediatric patients from a total of 516 with skull base chordomas. The craniocervical junction was deemed as the most common location (n=33), with the highest proportion of large tumors (27.3%, yet the lowest rate for gross total resection (GTR) (9.1%). This low GTR rate was deemed as statistically significant compared to chordomas in other locations [27].

## Conclusions

Neoplasms within the craniocervical junction of the skull base are a complex pathology that requires careful prognostic evaluation and management. Surgical resection has proven to be challenging due to anatomical constraints and the size of the patient. Future implementation of robotic or hybrid techniques may be warranted for improving outcomes. Among the meningiomas, schwannomas, and chordomas, the latter have shown the most versatile management methods with incorporation of molecular targets, warranting the investigation of future therapies to include pathophysiological inhibitors. Biomarker analysis should be further established for predictors of poor outcomes and early interventions. Nevertheless, the surgical management of pediatric neoplasms at the CCJ has indicated positive outcomes, prolonging pediatric mortality and preserving the quality of life.
